# Effectiveness of Negative Pressure Wound Therapy With Instillation and Dwell in Removing Nonviable Tissue, Promoting Granulation Tissue, and Reducing Surgical Debridements: A Systematic Literature Review

**DOI:** 10.1111/wrr.70059

**Published:** 2025-06-30

**Authors:** Julie Acosta, Lydia Galarza, Margaret Marsh, Ricardo R. Martinez, Mark Eells, Ashley W. Collinsworth

**Affiliations:** ^1^ Medical Surgical Solventum Maplewood, MN USA

**Keywords:** debridement, negative pressure wound therapy with instillation, wound cleansing, wound treatment

## Abstract

Surgical debridement is a common treatment for complex wounds but can present risks for patients. Negative pressure wound therapy with instillation and dwell (NPWTi‐d) using reticulated open cell foam dressings with 1 cm holes (ROCF‐CC) provides hydromechanical wound cleaning and preparation and can be applied outside the operating room at the bedside. This systematic literature review examined the effectiveness of NPWTi‐d with ROCF‐CC in removing nonviable tissue and infectious material, promoting granulation tissue, and reducing surgical debridements. A systematic search was conducted utilising PubMed, Embase, and ClinicalTrials.gov to identify studies conducted from 1 January 2015–31 August 2022. Study outcomes related to nonviable tissue, granulation tissue, and debridement were summarised and analysed using descriptive statistics. Twenty‐one studies including 178 patients who received NPWTi‐d with ROCF‐CC were included. Evidence of reduction in necrotic and infected tissue following treatment was observed in 97.9% of wounds across 17 studies. Formation of granulation tissue after NPWTi‐d with ROCF‐CC was reported in 99.2% of wounds across 14 studies. Over 63% of patients avoided surgical debridements in 8 studies, and a statistically significant decrease in surgical debridements was noted in 2 comparative studies. This systematic review provides real‐world evidence demonstrating the effectiveness of NPWTi‐d with ROCF‐CC in the hydromechanical removal of infectious materials, non‐viable tissue, and wound debris; reduction of surgical debridements; and promotion of granulation tissue. Thus, NPWTi‐d with ROCF‐CC may potentially reduce or eliminate the need for surgical debridement by removing non‐viable tissue through hydromechanical action.

**Abbreviations**
NPWTi‐d: negative pressure wound therapy with instillation and dwell
ROCF‐CC: reticulated open cell foam dressings with 1 cm holes

## 1 Introduction

Debridement is an important technique in the treatment of complex wounds and is defined as the removal of any non‐viable material or debris, such as necrotic tissue, slough, infectious material, biofilm, and foreign bodies from the wound bed to promote wound healing [1, 2, 3]. This definition applies to various methods of debridement including autolytic, biological, enzymatic, surgical/sharp, mechanical, chemical, hydrosurgical, or ultrasonic. Surgical or sharp debridement is an effective method commonly used in wound care. However, there are often reasons why this procedure may not be available or appropriate, such as the patient's poor general condition, the risk of excessive damage which can delay healing, risk of bleeding, or risks of anaesthesia [4].

**FIGURE 1:**
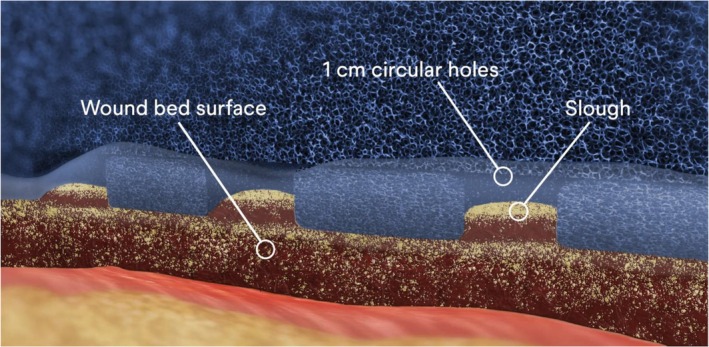
Negative pressure wound therapy with topical wound solution instillation and dwell (NPWTi‐d) used in conjunction with reticulated open cell foam with through holes (ROCF‐CC). Used and reprinted with permission from Solventum.

Negative pressure wound therapy with topical wound solution instillation and dwell (NPWTi‐d) used in conjunction with reticulated open cell foam with 1 cm holes (ROCF‐CC) can facilitate non‐viable tissue extraction (Figure 1) and can be a bedside alternative to surgical debridement. NPWTi‐d is an advancement over traditional NPWT in that it features 2 wound treatments in one device. Extraction of infectious materials, non‐viable tissue, and wound debris is achieved through the dressing's mechanical action in conjunction with the process of instilling and allowing topical solutions to soak in the wound bed for up to 30 min during the instillation phase. Thus, NPWTi‐d with ROCF‐CC may potentially eliminate the need for or reduce the frequency or extent of surgical debridement when required.

There is a growing body of evidence demonstrating the effectiveness of NPWTi‐d as an adjunctive therapy for a variety of infected, contaminated, or complex wounds [5, 6, 7, 8]. Use of NPWTi‐d has been associated with improved patient outcomes such as reduced time to final surgical procedure and hospital length of stay, and increased likelihood of wound closure before discharge compared to NPWT alone [6, 7, 8]. Although there is growing evidence that use of NPWTi‐d can facilitate overall wound healing, no large comparative studies have focused on the effectiveness of NPWTi‐d with ROCF‐CC in providing hydromechanical wound debridement. The objective of this systematic review was to assess the real‐world evidence of the effectiveness of NPWTi‐d with ROCF‐CC in removing nonviable tissue and infectious material, promoting granulation tissue formation, and reducing the need for surgical debridement.

## 2 Materials and Methods

A systematic literature review was conducted using an internal protocol that conformed to PRISMA guidelines [9] to identify studies that examined the effectiveness of NPWTi‐d with ROCF‐CC in providing hydromechanical removal of non‐viable and infectious materials for wounds. The primary outcome of interest was a reduction in non‐viable tissue, such as slough, fibrotic tissue, necrotic tissue, or other unspecified non‐viable tissue. Other key outcomes included the number of surgical debridements and promotion of granulation tissue formation. All other reported outcomes related to the effectiveness of NPWTi‐d with ROCF‐CC in promoting wound healing were documented.

### 2.1 Literature Review

A search of published studies was conducted utilising PubMed and EMBASE. The following search string was used: ((‘Negative Pressure Wound Therapy’ OR ‘Vacuum Assisted Closure’ OR ‘Vacuum Sealing’ OR ‘topical negative pressure’ OR ‘negative pressure therapy’ OR ‘subatmospheric pressure’ OR ‘sub‐atmospheric pressure’ OR ‘NPWT’ OR ‘NPWTi’ OR ‘NPWTi‐d’ or ‘NPWTid’ OR ‘NPWT‐i’ OR ‘NPWT‐id’ OR ‘Ulta’ OR ‘VERAFLO’ OR ‘VERAFLOW’) AND (‘Lavage’ OR ‘Instill’ OR ‘Instillation’ OR ‘Irrigate’ OR ‘Irrigation’ OR ‘Topical Solution’ OR ‘Topic Solution’ OR ‘Topical wound solution’ OR ‘VERAFLO’ OR ‘VERAFLOW’ OR ‘VERAFLO CLEANSE DRESSING’ OR ‘VERAFLO CLEANSE CHOICE DRESSING’ OR ‘CLEANSE CHOICE COMPLETE’ OR ‘ULTA’ OR ‘ROCFCC’ OR ‘ROCF‐CF’ OR ‘ROCF‐CCC’ OR (‘open cell foam’ AND ‘through holes’)) AND (‘debridement’ OR ‘debride’ OR ‘non‐viable’ OR ‘nonviable’ OR ‘non viable’ OR ‘necrotic tissue’ OR ‘necrosis’ OR ‘fibrotic tissue’ OR ‘slough’ OR ‘solubilise’ OR ‘soften’)).

In addition, a search of registered studies was conducted using ClinicalTrials.gov to identify completed trials with unpublished study data using the following search string: (‘VERAFLO’ OR ‘VERAFLOW’ OR ‘VERAFLO CLEANSE DRESSING’ OR ‘VERAFLO CLEANSE CHOICE DRESSING’ OR ‘CLEANSE CHOICE COMPLETE’ OR ‘ULTA’ OR ‘ROCF‐CC’ OR ‘ROCF‐CF’ OR ‘ROCF‐CCC’ OR ‘INSTILLATION’).

Peer‐reviewed manuscripts, conference posters, and abstracts that were published in English from 1 January 2015—31 August 2022, featuring one manufacturer's NPWTi‐d device (3M Veraflo Therapy, Solventum Corporation, Maplewood, MN) and ROCF‐CC (3M Veraflo Cleanse Choice Dressing or 3M Veraflo Cleanse Choice Complete Dressing, Solventum Corporation, Maplewood, MN) were eligible for study inclusion. Other inclusion criteria were outcomes related to debridement, removal of necrosis; removal or decrease of non‐viable tissue, necrotic tissue, fibrotic tissue or slough; debridement; or terms including ‘solubilise’, ‘soften’ or ‘debride’. Studies also had to include information and data points pertaining to each patient's wound before and after use of NPWTi‐d with ROCF‐CC to directly compare wound conditions at initiation of therapy to conditions throughout and after treatment. For studies with multiple publications, only the publication with the most complete data related to the outcomes of interest was included. Meta‐analyses, systematic reviews, protocols, pre‐clinical studies, veterinary studies, and single‐patient studies were excluded as were studies that featured off‐label use, lacked safety or performance data, or did not have a finalised study report in ClinicalTrials.gov.

An initial screening of titles and abstracts was conducted by one reviewer to remove duplicates and studies not meeting inclusion criteria. Two independent reviewers conducted full‐text reviews of the remaining studies to determine whether they met all the inclusion criteria and none of the exclusion criteria. When there was a disagreement between the reviewers regarding inclusion, a third reviewer reviewed the study and served as the tie‐breaker.

### 2.2 Data Extraction, Synthesis, and Analysis

Information related to the studies was documented by reviewers in an Excel spreadsheet. Extracted information included country of origin, if the study was peer‐reviewed, study type, total patients, wound types, patient characteristics, number of patients receiving NPWTi‐d with ROCF‐CC and control therapies, type of control therapy, NPWTi‐d settings and instillation solution, study objectives, study performance outcomes, safety outcomes, and study conclusions. Study outcomes were grouped into categories related to nonviable tissue, granulation, debridement, and other wound outcomes and analysed using descriptive statistics. Numerators and denominators were summed across studies that reported the same binary outcomes. For studies with comparative treatment groups that assessed the same outcomes, combined study means and medians were calculated and reported.

### 2.3 Risk of Bias

Studies that met the inclusion and exclusion criteria were evaluated for risk of bias and appropriateness for inclusion in the literature review using a process for clinical evaluation developed with guidance from the Global Harmonisation Task Force [10]. The appraisal criteria included measures of suitability, data contribution, and level of evidence. Studies were scored based on these criteria and a weighting score and percent were calculated for each study to indicate how well the clinical data assessed the effectiveness of NPWTi‐d with ROCF‐CC.

## 3 Results

### 3.1 Results of the Literature Search

**FIGURE 2:**
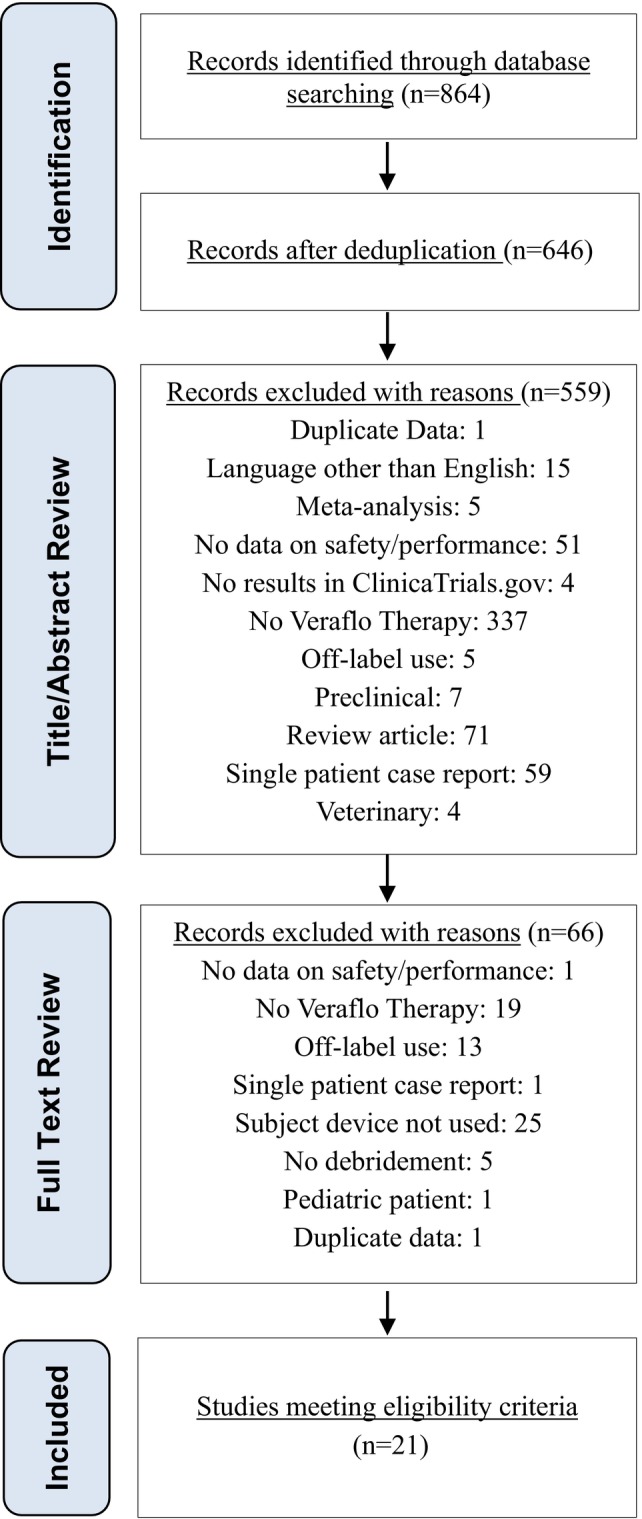
Results of the literature search.

A total of 864 studies were identified through the search of the published literature and ClinicalTrials.gov (Figure 2). After the removal of duplicates, the titles and abstracts of 646 studies were reviewed for potential inclusion. Eighty‐seven studies met the criteria for a full‐text review with 21 studies meeting the inclusion criteria for the systematic review.

### 3.2 Study Characteristics

Characteristics of the 21 included studies are described in Table 1. Most of the studies were conducted in the United States (*n* = 16). The other studies were conducted in New Zealand, Japan, Canada, and France, and one publication included case studies from various locations in Europe. Eighteen of the studies were case series and 3 of the studies were retrospective comparative studies. A total of 178 wounds were treated with NPWTi‐d with ROCF‐CC in the included studies (Table 2). The most common wound types were surgical/dehisced wounds (43.8%) and pressure ulcers/injuries (27.5%). Other wound types included traumatic wounds, diabetic foot wounds, chronic wounds, burns, and venous leg ulcers. Patients ranged in age from 34 to 98 years and had a variety of comorbidities.

**TABLE 1:** Study characteristics.

Author	Country	Peer‐reviewed	Study type	# of patients	Age, mean (range)	Control therapy	Wound types	NPWTi‐d settings	ROCF dressing	Instillation solution	Nonviable tissue outcomes	Granulation outcomes	Debridement outcomes	Other wound outcomes
Aburn [11]	New Zealand	Yes	CS	I: 5 C: 0	64.4 (46–85)	N/A	Vascular patients with complex wounds	Dwell time 20 min NPWT time 3.5 h (4) 5 h (1) Pressure −125 mmHG Duration (mean) 7 days	CC	Prontosan wound irrigation solution	Improved wound bed preparation shown with silhouette photography I: 5 (100%)	NR	Avoided surgical debridement I: 5 (100%)	Significant pain reduction I: 4 (80%)
Blalock [12]	USA	Yes	CS	I: 19 C: 0	57.1 (24–87)	N/A	Surgical (8) Trauma (4), Pressure injury (4), DFU (1) Arterial ulcer (1), Non‐pressure chronic ulcer (1)	Dwell time 1–10 min NPWT time 2–3.5 h Pressure −125 or −150 mmHG Duration (mean) 9 days	CC	0.025% Dakin's solution	Reduction in devitalized tissue I: 19 (100%)	Improved granulation I: 19 (100%)	NR	High risk patients avoiding amputation I: 2/2 (100%)
Chowdhry [13]	USA	No	CS	I: 4 C: 3	40.0 (NR)	NPWTi‐d with non‐reticulated foam dressing	Post‐surgical and trauma wounds	Dwell time 20 min NPWT time 2 h Pressure −125 mmHG Duration 6–10 days	CC	Dakin's solution (1/8 strength)	NR	NR	Wound surface needing debridement (cm^2^) Before NPWTi‐d I and C: 243.4 ± 117.4 After NPWTi‐d I and C: 74.3 ± 20.9	Complete wound closure I: 4 (100%) C: 3 (100%)
Chowdhry [5]	USA	Yes	RC	I: 15 C: 15	70.0 (NR)	Wet‐to‐moist dressings soaked in 1/8 strength Dakin's solution	Sternal wounds that failed to heal	Dwell time 20 min NPWT time 2 h Pressure −125 mmHG Duration 3–10 days	CC	Dakin's solution (1/8 strength)	NR	NR	Debridements/dressing changes, mean (SD) I: 1.8 (0.7) C: 3.1 (1.0), *p* = 0.0011	Time to wound closure (days) I: 7.9 ± 2.3 C: 13.9 ± 3.2, *p* < 0.0001 Length of therapy (days) I: 5.4 ± 2.1 C: 8.4 ± 2.9, *p* = 0.0041 Drain duration (days) I: 15.0 ± 2.0 C: 21.7 ± 3.9, *p* = 0.0001
Cole [14]	USA	Yes	RC	I: 5 C: 5	69.8 (54–88)	Advanced wound dressings	DFU (4) VLU (2) Pressure injury (2) Trauma wound (1) Hematoma (1)	Dwell time 20 min NPWT time 2 h Pressure −125 mmHG Duration NR	CC	Normal saline	NR	NR	Debridements I: 1.00 ± 0.00 C: 7.00 ± 10.1, *p* = 0.004	Wound closure I: 5/5 (100%) C: 2/5 (40%)
Delapena [15]	USA	Yes	CS	I: 10 C: 0	57.3 (31–78)	N/A	Necrotizing soft tissue infections (6) Sacral pressure injury (2) Burn injuries (2)	Dwell time 15–30 min NPWT time 3 h Pressure NR Duration NR	CC	HOCl solution	Reduction in necrotic and infected tissue I: 10 (100%)	NR	Debridements Before NPWTi‐d, mean I: 6.2 After NPWTi‐d, mean I: 0	Wound closure I: 9/10 (90%)
Dingess [16]	USA	No	CS	I: 3 C: 0	NR (54–80)	N/A	Foot ulcer (1) VLU (1) Trauma (1)	Dwell time 10 min NPWT time 3.5 h Pressure −125 mmHG Duration 4–7 days	CC	Normal saline	Removal of exudate and infectious materials I: 3 (100%)	NR Case photos do provide visual evidence of granulation tissue formation in all 3 cases	NR	Complete wound healing I: 3
Fernandez [17]	USA	Yes	CS	I: 19 C: 0	58.2 NR	N/A	Pressure injuries (7) Traumatic (5) Surgical (3) Soft tissue infection (3) Chronic (1)	Dwell time 5–10 min NPWT time 2–3.5 h Pressure −125 mmHG Duration 9.5 ± 4.1 days	CC	Quarter‐strength Dakin's solution, HOCl, or normal saline	Reduction of slough and exudate within 72 h I: 19/19 (100%)	Increase in granulation tissue within 72 h I: 19/19 (100%)	NR	NR
Fernandez [18]	USA	Yes	CS	I: 5 C: 0	65.2 (50–82)	N/A	Pressure injuries	Dwell time 10 min NPWT time 2–3 h Pressure −125 mmHG Duration 2–9 days	CC	Normal saline or HOCl	Improved removal of devitalized tissue in patients receiving HOCl compared to saline	Increase in granulation I: 5 (100%) Improved granulation tissue in patients receiving HOCl compared to saline	NR	NR
Fukui [4]	Japan	Yes	CS	I: 4 C: 0	NR (66–74)	N/A	Necrotizing fasciitis (1) Surgical (1) Ulcer (1) DFU (1)	Dwell time 5–10 min NPWT time 2–3.5 h Pressure −125 mmHG Duration 8–28 days	CC	Normal saline	Removal of necrotic tissue I: 4 (100%)	Increase in granulation tissue I: 4 (100%)	Debridement could be easily performed with cupped forceps following NPWTi‐d	Wound closure I: 4 (100%), 3 via grafting, 1 with ointment therapy
Hill [19]	Canada	No	CS	I: 3 C: 3	NR (58–89)	ciNPT over closed incisions	Hematoma excision (1) Pressure ulcer (1) Surgical wound from abdominal perineal resection (1) Closed incisions of the perineal region (controls) (3)	Dwell time 5 min NPWT time 2–3 h Pressure −125 mmHG Duration 10–14 days	CC	Normal Saline	Removal of infectious material I: 3 (100%)	NR Case photos do provide visual evidence of granulation tissue formation in 1 case	Avoided surgical debridement I: 3 (100%)	NR
Kalos [20]	USA	No	CS	I: 4 C: 0	NR (49–79)	N/A	Pressure injury (2) Infected surgical (1) DFU (1)	Dwell time 3–10 min NPWT time 3–4 h Pressure −75 to −125 mmHG Duration 8 ± 2 days	CC	Normal Saline or HOCl	Reduction in necrotic and infected tissue I: 4 (100%)	Development of healthy granulation tissue I: 4 (100%)	Avoided surgical debridement I: 3 (75%)	Patients transitioning to conventional NPWT I: 4 (100%)
Klein [21]	USA	Yes	CS	I: 4 C: 0	NR (65–95)	N/A	DFU (2) Dehisced surgical (1) Neuropathic ulcer (1)	Dwell time 1 min NPWT time 3 h Pressure −125 mmHG Duration 3–16 days	CC	Normal Saline	Reduced slough I: 4 (100%)	Increased granulation I: 4 (100%)	NR	Closure achieved 4 (100%)
Matthews [22]	USA	Yes	CS	I: 5 C: 0	NR (55–87)	N/A	Burns (4) Necrotizing fasciitis (1)	Dwell time 20–30 min NPWT time 3 h Pressure −125 mmHG Duration NR	CC	Vashe (HOCl)	Decrease in necrotic material I: 5 (100%)	Improved granulation I: 5 (100%)	NR	Wounds closed with split thickness skin graft I: 4 (80%) STSG pending for remaining patient
McElroy [23]	USA	Yes	CS	I: 14 C: 0	63.6 (NR)	N/A	Pressure injury (3) Necrotizing fasciitis (2) DFU (2) Surgical (4) Other/unknown (3)	Dwell time 2–10 min NPWT time 0.5–4 h Pressure −125 mmHG Duration 1–15 days	CC	Normal Saline, acetic acid, or HOCl solution	Decrease in devitalized tissue I: 14 (100%)	Improved granulation I: 14 (100%)	Avoided surgical debridement I: 12 (85.7%)	NR
McElroy [24]	USA	No	CS	I: 3 C: 0	NR (36–72)	N/A	Chronic ulcer (1) Necrotizing fasciitis (1) Cellulitis (1)	Dwell time 2–10 min NPWT time 1–3.5 h Pressure −125 mmHG Duration 10–22 days	CC	Normal Saline or HOCl solution (0.125%)	Reduction in necrotic and infected tissue I: 3 (100%)	Improved granulation I: 3 (100%)	NR	NR
Multiple Authors [25]	Europe	No	CS	I: 5 C: 0	NR (61–81)	N/A	DFU with gangrene (1) Necrotizing fasciitis (2) VLU (1) Calciphylaxis ulcer (1)	Dwell time 10–15 min NPWT time 2–3.5 h Pressure −125 mmHG Duration 13–49 days	CC	Normal Saline, Polyhexanide solution (0.04%), physiological solution +0.05% sodium hypochlorite	Reduction in nonviable tissue I: 5 (100%)	Increase in granulation tissue I: 5 (100%)	Avoided surgical debridement I: 4 (80%)	Wound bed prepared for closure I: 3 (60%) Reduction in wound size I: 1 (20%) Avoided amputation I: 3 (60%)
Obst [26]	USA	No	RC	I: 9 C: 11	56.1 (NR)	NPWT (historical)	Abdominal surgical wounds	Dwell time 3–10 min NPWT time 2–3.5 h Pressure −100 or −125 mmHG Duration 5–8 days (4) > 8 days (2)	CC	Normal Saline	Development of nonviable tissue I: 0 (0%) C: 11 (100%)		Patients requiring at least 1 debridement after NPWT I: 0 (0%) C: 11 (100%); *p* = 0.001	Number of days to wound healing
Obst [27]	USA	Yes	CS	I: 6 C: 0	NR (34–70)	N/A	Large abdominal wound (1) Necrotizing soft tissue infection (1) Pressure injuries (3) Perianal abscess (1)	Dwell time 3–10 min NPWT time 2–3.5 h Pressure −100 or −125 mmHG Duration 5–8 days (4) > 8 days (2)	CC	Normal Saline or ¼ strength sodium hypochlorite solution	Reduction in necrotic and infected tissue I: 6 (100%)	Improved granulation tissue I: 6 (100%)	NR	Wound bed prepared for closure I: 6 (100%)
Teot [28]	France	Yes	CS	I: 21 C: 0	55.4 NR	N/A	Pressure injuries (18) Burns (1) Necrosis after skin excision (2)	Dwell time 10 min NPWT time 3–5 h Pressure −125 mmHG Duration 8–7 days	CC	Normal Saline	Reduction in surface area of black non‐viable tissue to ≤ 10% I: 18/21 (85.7%) Reduction in surface area of yellow fibrinous slough to ≤ 10% I: 12/21 (57.1%)	Development of healthy granulation tissue I: 20 (95.2%)	Avoided surgical debridement I: 10 (47.6%)	NR
Willmore [29]	USA	Yes	CS	I: 15 C: 0	69 (56–98)	N/A	Surgical dehiscence (7) Hard‐to‐ heal wound (2), Pressure injury (2) DFU (2), Cat bite (1) Amputation left open (*n* = 1)	Dwell time 10 min NPWT time 15 min Pressure −125 mmHG Duration 4–36 h	CC	Normal Saline or 0.125% sodium hypochlorite solution	Reduction in necrotic and infected tissue I: 15 (100%)		Avoided surgical debridement I: 2 (13.3%)	Reduced edema I: 15 (100%) Reduced erythema in peri‐wound area I: 15 (100%) Reduced WBC count, (mean) I: −2500 ± 800 cells/μL *p* < 0.001

Abbreviations: C, control group; CC, 3M Veraflo Cleanse Choice Dressing; ciNPT, closed incision negative pressure therapy; CS, case series; HOCl, hypochlorous acid; I, intervention group; NR, not reported, RC, retrospective comparative; VLU, venous leg ulcer; WBC, white blood cell.

**TABLE 2:** Wound types in the included studies.

Wound type	*N* (%)
Surgical/dehisced	78 (43.8)
Pressure ulcers/injuries	49 (27.5)
Traumatic	14 (7.9)
Diabetic foot ulcer	12 (6.7)
Chronic	10 (5.6)
Burn	7 (3.9)
Venous leg ulcer	4 (2.2)
Other/unknown	4 (2.2)
Total	178 (100.0)

### 3.3 NPWTi‐d Settings and Dressings

Most studies followed recommended use of NPWTi‐d with ROCF‐CC, instilling saline or a hypochlorous acid solution with a 1‐ to 10‐min dwell time followed by 2–3.5 h of negative pressure (−125 or −150 mmHg). Longer dwell times of 15–30 min [5, 11, 13, 14, 15, 22, 25] and NPWT cycles of 4–5 h [11, 20, 23, 28] were reported for some patients in some of the studies. One study reported a shorter cycle of NPWT of 15 min, with NPWTi‐d continued for a brief period of 4–36 h or until the OR became available [29]. Some studies also reported negative pressure settings of −75 mmHg [20] or −100 mmHg [26, 27] for some patients. Dressing changes were performed every 2–3 days and duration of NPWTi‐d was based on wound size and other patient factors. Duration of NPWTi‐d with ROCF‐CC varied among the included studies and ranged from 4 h to 49 days, although many studies reported a treatment duration ranging from 3 to 10 days. Three studies did not report on duration of therapy [14, 15, 22]. NPWTi‐d was used with 3 M Veraflo Cleanse Choice ROCF‐CC dressings in all patients who received the therapy.

### 3.4 Study Outcomes

Seventeen of the studies reported on the primary outcome of reduction in necrotic and infected tissue (Tables 1 and 3) [4, 11, 12, 15, 16, 17, 18, 19, 20, 21, 22, 23, 24, 25, 27, 28, 29]. Of the 145 wounds that were treated with NPWTi‐d with ROCF‐CC in these case studies, 142 (97.9%) demonstrated a reduction in necrotic and infected tissue following treatment. In one study, a reduction in necrotic tissue was not observed in one patient, and one patient had NPWTi‐d discontinued due to pain at dressing changes, which also occurred with prior treatment [11]. However, improved wound bed reparation was observed following therapy in both cases, and both patients avoided surgical debridement [11]. NPWTi‐d with ROCF‐CC was discontinued early due to a deep tissue infection unrelated to the therapy in one patient in another study [28].

**TABLE 3:** Clinical outcomes associated with use of NPWTi‐d with ROCF.

Clinical outcomes	Studies with reported outcome (*N* = 21)	Treatment group patients	Control group patients
Nonviable tissue outcomes			
Reduction in necrotic and infected tissue [4, 11, 12, 15, 16, 17, 18, 19, 20, 21, 22, 23, 24, 25, 27, 28, 29]	17	142/145 (97.9%)	NR
Prevention of nonviable tissue buildup [26]	1	9/9 (100%)	0/11 (0%)
Granulation outcomes			
Granulation tissue formation [4, 5, 12, 14, 17, 18, 20, 21, 22, 23, 24, 25, 27, 28]	14	128/129 (99.2%)	NR
Debridement outcomes			
Avoided surgical debridement [11, 19, 20, 23, 25, 26, 28, 29]	8	48/76 (63.1%)	0/11 (0%)
Significantly fewer surgical debridements, (combined median) [5, 14]	2	1.5	3.0
Reduced wound surface area requiring debridement [13]	1	4/7 (57.1%)	NR
Other wound outcomes			
Prepared wound bed for closure [11, 13, 15, 19, 21, 25, 27]	7	33/40 (82.5%)	NR
Complete wound closure/healing [11, 13, 14, 15, 16, 19, 21, 25, 27]	9	42/48 (87.5%)	2/5 (40.0%)
Shorter time to wound closure (days, combined mean, SD) [5, 26]	2	28.7 (37.9)	77.3 (102.3)
Reduction in wound size [11, 25]	2	2/10 (20%)	NR
Fewer days of therapy, days (median) [5]	1	6	8
Shorter drain duration, days (median) [5]	1	14	22
Wounds remaining healed at 90 days [5]	1	15/15 (100%)	15/15 (100%)
Pain reduction [11]	1	4/5 (80.0%)	NR
Decreased edema [29]	1	15/15 (100%)	NR
Decreased erythema [29]	1	15/15 (100%)	NR
Avoidance of amputation [12, 25]	2	5/7 (71.4%)*	NR

Abbreviation: NR, not reported.

* Limited to patients where risk of amputation was noted.

Additionally, one comparative study examined nonviable tissue buildup in patients with abdominal wounds receiving NPWTi‐d with ROCF‐CC compared to patients receiving NPWT without instillation [26]. None of the 9 patients who received NPWTi‐d with ROCF‐CC experienced nonviable tissue buildup while all 11 patients who received NPWT without instillation developed nonviable tissue (*p* < 0.001) (Tables 1 and 3) [26].

Fourteen of the included studies reported on the effect of NPWTi‐d with ROCF‐CC on the promotion of tissue granulation (Tables 1 and 3) [4, 5, 12, 14, 17, 18, 20, 21, 22, 23, 24, 25, 27, 28]. Of the 129 wounds included in these case studies, granulation tissue formation was observed following NPWTi‐d with ROCF‐CC in 128 (99.2%) of the wounds. Although the remaining 7 publications did not report granulation tissue outcomes per case, there were general statements supporting this benefit and/or visible evidence in the case photographs included in the publications.

Several of the included studies examined outcomes related to debridement (Tables 1 and 3). Approximately 63% of patients were able to avoid surgical debridement following the use of NPWTi‐d with ROCF‐CC across the 8 studies that examined this measure [11, 19, 20, 23, 25, 26, 28, 29]. Two comparative studies reported on the number of debridements [5, 14]. Chowdhry et al. found that patients with sternal wound complications who received NPWTi‐d with ROCF‐CC had significantly fewer debridements (2 vs. 3 (median), *p* = 0.0011) than patients who received wet‐to‐moist dressings [5]. Likewise, in a small study of patients with complex lower extremity wounds, Cole et al. observed that patients who received NPWTi‐d with ROCF‐CC had significantly fewer debridements (1 vs. 3 (median), *p* = 0.004) compared to patients who received advanced wound dressings (Table 3) [14].

Other clinical benefits observed with the use of NPWTi‐d with ROCF‐CC (Tables 1 and 3) included preparation of the wound bed for closure [11, 13, 15, 19, 21, 25, 27], complete wound closure [11, 13, 14, 15, 16, 19, 21, 25, 27], shorter time to wound closure [5, 26], reduction in wound size [11, 25], fewer days of therapy [5], shorter drain duration [5], all wounds remaining healed at 90 days [5], pain reduction [11], decreased edema [29], decreased erythema [29], and avoidance of amputation [12, 25].

The occurrence of complications and/or adverse events was examined in the 21 studies that met eligibility criteria (Table 4). In each of the comparative studies, there were no reported adverse events in the treatment groups while there were several complications reported in the control groups including seromas [5], need for at least one debridement to remove non‐viable tissue following NPWT [26], return to surgery for debridement [14], continued infection [14], toe amputation and trans‐metatarsal amputation [14], and a wound that remained open for > 2 years [14]. In publications where there was no control group, the reported adverse events included complications common to wound care and debridement techniques such as stalled wound healing not related to therapy [28], pain with dressing changes [4, 11, 25, 28], and maceration of peri‐wound tissue [4]. In 14 of the 21 publications, there were no adverse events reported for either patients treated with the subject device (114 cases) [12, 13, 15, 16, 17, 18, 20, 21, 22, 23, 24, 27, 29], or control therapy (3 cases) [19].

**TABLE 4:** Reported complications and adverse events.

Complications/adverse events	Number of studies (*N* = 21)	Treatment group patients *N* (%)	Control group patients *N* (%)
Seroma [5]	1	0/15 (0)	3/15 (20.0)
Required at least one debridement to remove non‐viable tissue following NPWT [26]	1	0/9 (0)	11/11 (100.0)
Return to surgery for debridement [14]	1	0/5 (0)	2/5 (40.0)
Continued infection [14]	1	0/5 (0)	1/5 (20.0)
Toe amputation, TMA [14]	1	0/5 (0)	1/5 (20.0)
Wound open for > 2 years [14]	1	0/5 (0)	1/5 (20.0)
Pain with dressing changes [4, 11, 25, 28]	4	11/35 (31.4)	N/A
Patient with diabetic ulcer experienced maceration of the peri‐wound skin [4]	1	1/4 (25.0)	N/A
No complications/adverse events reported [12, 13, 15, 16, 17, 18, 19, 20, 21, 22, 23, 24, 27, 29]	14	0/114 (0)	0/3 (0)

## 4 Discussion

This systematic literature review offers supporting real‐world evidence across a variety of complex wound types that NPWTi‐d with ROCF‐CC can provide hydromechanical removal of infectious materials, non‐viable tissue, and wound debris, which reduces the number of surgical debridements required, whereas promoting granulation tissue formation and creating an environment that promotes wound healing. Thus, NPWTi‐d with ROCF‐CC may provide an opportunity to eliminate the need for surgical debridement or reduce the frequency or extent of surgical debridement when necessary, thus avoiding or minimising the risks associated with this procedure.

Seventeen of the 21 included studies provided direct evidence of the ability of NPWTi‐d with ROCF‐CC to reduce necrotic and infected tissue following treatment as 97.9% of the 145 patients included in these studies had documented reductions in nonviable tissue and infectious material following treatment. In 14 of the 21 publications, promotion of granulation tissue formation following the use of NPWTi‐d with ROCF‐CC was directly reported in 128 of 129 cases. Although the remaining 7 publications did not report granulation tissue outcomes per case, there were general statements supporting this benefit and/or visible evidence in the case photographs included in the publications. Over 63% of patients were able to avoid surgical debridements in the 8 studies that reported this measure. Additionally, 2 included comparative studies examined the difference in the number of debridements and found that patients treated with NPWTi‐d with ROCF‐CC had significantly fewer debridements than patients treated with other dressings. In total, 177 out of the 178 patients in the included studies demonstrated direct or indirect evidence of improved wound bed preparation following NPWTi‐d with ROCF‐CC as indicated by reductions in necrotic or infected tissue, improvements in granulation, and/or the elimination or reduction of surgical debridements. Other positive outcomes were associated with the use of NPWTi‐d with ROCF‐CC in some studies including preparation of the wound bed for closure, complete wound closure, shorter time to wound closure, reduction in wound size, fewer days of therapy, shorter drain duration, wounds that remained healed at 90 days, pain reduction, decreased edema, decreased erythema, and avoidance of amputation.

The many positive outcomes observed following the use of NPWTi‐d with ROCF‐CC far outweighed the low numbers of reported complications or adverse events that are commonly associated with wound care. Pain with dressing changes was the most frequent complication and was reported by 11 patients. Téot et al. observed that a duration of therapy of more than 9 days appeared to result in enhanced growth of tissue into the dressing and caused pain at dressing changes for 6 of these patients [28]. The investigators decided to discontinue NPWTi‐d after 3 dressing changes, and only 1 patient reported pain following this protocol change [28]. Other authors noted that pain with dressing changes could be reduced by soaking the wound in saline or administering lidocaine before removing the foam dressing [4] or with the use of inhaled penthorox [11].

Use of NPWTi‐d with ROCF‐CC may spare patients additional pain and complications by reducing the need for surgical debridements and reducing time to wound closure. Despite being a common and effective treatment, surgical debridement may result in over‐excision and wound damage and carries a risk of bleeding [4, 28]. Published guidelines have recommended alternate, gentler methods of debridement for wounds where non‐viable tissue demarcation does not extend deeper than the deep dermal layer or the wound bed is covered by fibrin or slough [30]. Additionally, undergoing anaesthesia is not appropriate or is ill‐advised for many patients [28], particularly as treated populations are increasing in age and comorbid conditions, and operating rooms are not always available for surgical debridement. Data indicate that non‐viable tissue extraction using NPWTi‐d with ROCF‐CC does not damage the healthy parts of wounds and may facilitate the detachment of necrotic tissue [4]. Pairing ROCF‐CC with NPWTi‐d allows for early initiation of NPWTi‐d in complex wounds, which has been associated with improved outcomes including reductions in surgical debridements, days until final operating room procedure, wound‐related readmissions, and health care costs [31]. Use of NPWTi‐d with ROCF‐CC may also reduce time to healing, which can help prevent chronic infections and reduce inpatient days [11].

This systematic review addresses a gap in the literature as there are currently no large studies demonstrating the effectiveness of NPWTi‐d with ROCF‐CC in the hydromechanical removal of necrotic and infected tissue, reduction of debridements, and promotion of granulation tissue. Previous studies have highlighted the effectiveness of NPWTi‐d when used with ROCF without through holes in facilitating the removal of wound debris and exudate compared to traditional NPWT [6, 7, 32]. The benefits of NPWTi‐d may be further enhanced through the use of ROCF‐CC dressings based on the evidence contained within this review. The included evidence is for one manufacturer's device (3M V.A.C. Ulta Therapy System, Solventum Corporation, Maplewood, MN) and ROCF‐CC (3M Veraflo Cleanse Choice Dressing). Future studies may include these dressings or explore questions such as what kinds of necrotic tissue the therapy is most effective in removing or how much residual necrotic tissue can be present at the initiation of NPWTi‐d with ROCF‐CC [28].

### 4.1 Limitations

This study had several limitations. The included studies were mostly small case studies and case series without comparators. However, these case reports, regardless of publication source, contain data that have been obtained from clinician health records for their wound care patients and are consistent with FDA's guidance for using real‐world evidence. In each study, data was collected on each patient's wound before and after the use of NPWTi‐d with ROCF‐CC allowing individual patients to serve as their own control and for direct comparisons of wound conditions at initiation of therapy to conditions throughout and after treatment. Although most of the studies were small, this review demonstrated the effectiveness of NPWTi‐d with ROCF for patients with heterogeneity in wound type, medical conditions/comorbidities, and demographics. There was a potential for selection bias, as the published case studies may have highlighted cases with favourable outcomes. Some studies were limited to qualitative wound assessments. The 3 comparative studies were small retrospective studies, with the largest having 15 patients per arm. In some cases, little to no details were provided about previous methods of wound management. There were likely differences in protocols for NPWTi‐d with ROCF‐CC use across facilities. Despite the limitations above, each included study consistently supports the ability of NPWTi‐d with ROCF‐CC to safely provide hydromechanical removal of infectious materials, non‐viable tissue, and wound debris and promotion of granulation tissue, which are key components of the wound healing process.

## 5 Conclusion

This systematic literature review provides real‐world evidence demonstrating the effectiveness of NPWTi‐d with ROCF‐CC in the hydromechanical removal of infectious materials, non‐viable tissue, and wound debris which may reduce the number of surgical debridements required, whereas promoting granulation tissue formation, creating an environment that promotes wound healing. Although existing studies related to this topic are limited to case series and a few small comparative studies, they consistently demonstrate reductions in necrotic and infectious material and improvements in granulation following use of NPWTi‐d with ROCF‐CC across a variety of wound types and patient populations. Thus, NPWTi‐d with ROCF‐CC may potentially reduce or eliminate the need for surgical debridement by removing non‐viable tissue through hydromechanical action outside the operating room at the patient's bedside. Additional studies, including prospective randomised controlled trials, are forthcoming and may provide more insight on the impact of NPWTi‐d with ROCF‐CC on wound‐related outcomes and examine the cost‐effectiveness of the therapy.

## Conflicts of Interest

All authors are Solventum employees and shareholders.

## Data Availability

No datasets were generated or analysed during the current study.
